# Anomalous Behavior of Hyaluronan Crosslinking Due to the Presence of Excess Phospholipids in the Articular Cartilage System of Osteoarthritis

**DOI:** 10.3390/ijms18122779

**Published:** 2017-12-20

**Authors:** Piotr Bełdowski, Piotr Weber, Tomasz Andrysiak, Wayne K. Augé, Damian Ledziński, Tristan De Leon, Adam Gadomski

**Affiliations:** 1 Institute of Mathematics and Physics, UTP University of Science and Technology, PL 85796 Bydgoszcz, Poland; piotr.beldowski@utp.edu.pl (P.B.); adam.gadomski@utp.edu.pl (A.G.); 2 Atomic and Optical Physics Division, Department of Atomic, Molecular and Optical Physics, Gdańsk University of Technology, PL 80233 Gdańsk, Poland; pweber@mif.pg.gda.pl; 3 Faculty of Telecommunications, Computer Science and Technology, UTP University of Science and Technology, PL 85796 Bydgoszcz, Poland; damian.ledzinski@utp.edu.pl; 4 Department of Research and Development, NuOrtho Surgical, Inc., Boston, MA 02723, USA; nnmoc@aol.com; 5 College of Mathematics, Natural Sciences and Technology, Delaware State University, Dover, DE 19901, USA; tristan.deleon12@gmail.com

**Keywords:** hyaluronic acid, phospholipids, lubrication, articular cartilage, molecular dynamics simulations

## Abstract

Lubrication of articular cartilage is a complex multiscale phenomenon in synovial joint organ systems. In these systems, synovial fluid properties result from synergistic interactions between a variety of molecular constituent. Two molecular classes in particular are of importance in understanding lubrication mechanisms: hyaluronic acid and phospholipids. The purpose of this study is to evaluate interactions between hyaluronic acid and phospholipids at various functionality levels during normal and pathological synovial fluid conditions. Molecular dynamic simulations of hyaluronic acid and phospholipids complexes were performed with the concentration of hyaluronic acid set at a constant value for two organizational forms, extended (normal) and coiled (pathologic). The results demonstrated that phospholipids affect the crosslinking mechanisms of hyaluronic acid significantly and the influence is higher during pathological conditions. During normal conditions, hyaluronic acid and phospholipid interactions seem to have no competing mechanism to that of the interaction between hyaluronic acid to hyaluronic acid. On the other hand, the structures formed under pathologic conditions were highly affected by phospholipid concentration.

## 1. Introduction

Hyaluronan (HA) is a major component in many biological systems, providing functions such as modulation of fluid viscosity, surfacial lubrication, and interstitial cell-based homeostasis [1]. This study focuses on the important tribological role HA plays in synovial joint organ systems during conditions when synovial fluid phospholipids (PL) are present in excess due to pathogenesis. Such condition has been notably observed in the varied phenotype expressions of osteoarthritis and, along with PL molecular species adaptations, bond saturation profile shifts, and altered synovial fluid pH, have confounded clinical treatment efforts designed to normalize the intra-articular environment [2,3].

While the source of excessive phospholipids is multifactorial, recent surgical repair advances have created conditions that decrease its biological burden in human biomechanical processes. Damaged articular cartilage surfaces lack a superficial active phospholipid layer which disrupts the efficiency of all lubrication’s regimes between juxtaposed articular cartilage surfaces.

Without a surface active phospholipid layer, the exposed interstitial matrix remains a site of abnormal wear allowing molecular extrusion, including excess phospholipids, into the synovial fluid, constituting a reservoir which increases the biological load of intra-articular molecular debris [4]. This biological load has been typically associated with the non-mechanical symptoms of degenerative joint disease. Prior to enabling surgical repair of the surface active phospholipid layer, efforts to normalize the intra-articular environment had remained unsuccessful.

**Figure 1:**
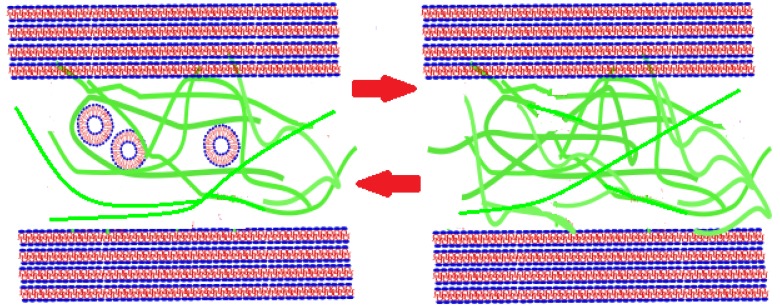
Artistic depiction of simplified composition articular cartilage in synovial joint organ system at different levels of functionality. The articular cartilage surfaces are depicted as multilamellar bilayers of the surface active phospholipid layer without the underlying cartilage zones. Micellar structures are shown between the articulating surfaces in various states of micellization and hyaluronan is shown as molecular chains (green) in various states of crosslinking and network formation. Donut-like structures represent PL vesicles. PL can create cylinder like structures surrounding HA chains and form more complex networks. Lubricin, water and other SF components have not been depicted in the picture.

Figure 1 presents a depiction of a synovial joint organ system with an intact multilamellar surface active phospholipid layer at different levels of functionality. Two separate cases of AC functioning are presented: low and high contact between both molecular species. To some extent, this picture can show simplified various regimes of lubrication. Namely, mixed and hydrodynamic have higher number of PL contacts, whereas boundary exhibits less penetration.

In the unique boundary lubrication under which kinetic friction coefficients are invariant relative to factors that influence a fluid film, the superficial active phospholipid layer is deemed to be a sacrificial layer during certain perturbation events and which subsequently can reform after the perturbation has been dissipated [5]. This wound healing feature has been exploited in the development of surgical devices designed to repair articular cartilage surfaces to an endpoint suitable for reconstitution of the surface active phospholipid layer [5,6].

In addition to PL alterations in the synovial fluid, notwithstanding a decrease of its biological burden, osteoarthritic phenotypes further demonstrate changes in HA molecular profile, often discussed as chain length polydispersity. HA polydispersity is increased with a general shift toward shorter chain molecules [2,3]. While it has been determined that hyaluronan’s tribological efficacy is generally improved when allowed to crosslink into supramolecular structures, physiological changes occurring commensurate with osteoarthritis modify its network forming properties within the system [7,8,9,10]. Shorter chain molecules impair system function as PLs can interact with these hyaluronan molecules to inhibit physical crosslinking and the formation of structural networks necessary for effective tribological constructs in vivo [2,3].

The purpose of this study is to evaluate hyaluronan behavior in the presence of varying PL concentrations typically observed in an osteoarthritic synovial joint organ system. Using in-silica experiments on the YASARA software program, analytic models were designed to imitate a nanoscopic section of the fluid filled synovial joint cavity with an intact surface active phospholipid layer representing the articular cartilage surface and joint cavity boundary post-surgical repair. Two HA networks are analyzed each with the same length of the HA molecule, that of a normal and an abnormal synovial joint organ system. The distinction between the HA networks analyzed is the initial structural condition of the molecule. HA in healthy synovial fluid creates dense networks of interacting chains, thus the contact area HA:HA is high. For extended HA chains, the interactions within its own species is higher as those interactions would be prior to interactions with other molecular species. Pathological (coiled) structure is designed to show the HA degradation mechanism during OA. Higher activity of enzymes degenerating HA cause more random short chain networks interacting with PL. However, as we showed in [11], this result can be applied to to wider spectrum of HA species in agreement with other experimental data [12]. Presented study is the extension of previously published paper [3]. However, in this study, we are more focused on intermolecular-intramolecular polymer network creation in the presence of phospholipids.

## 2. Results

**Figure 2:**
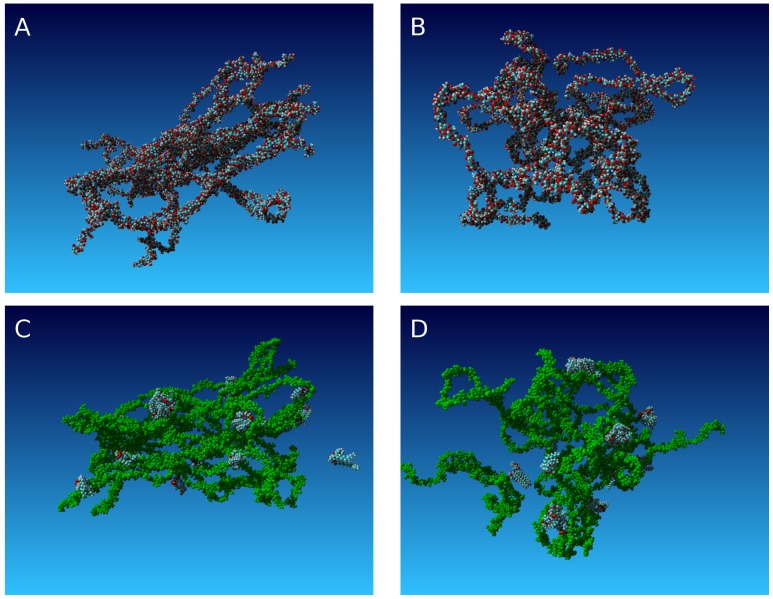

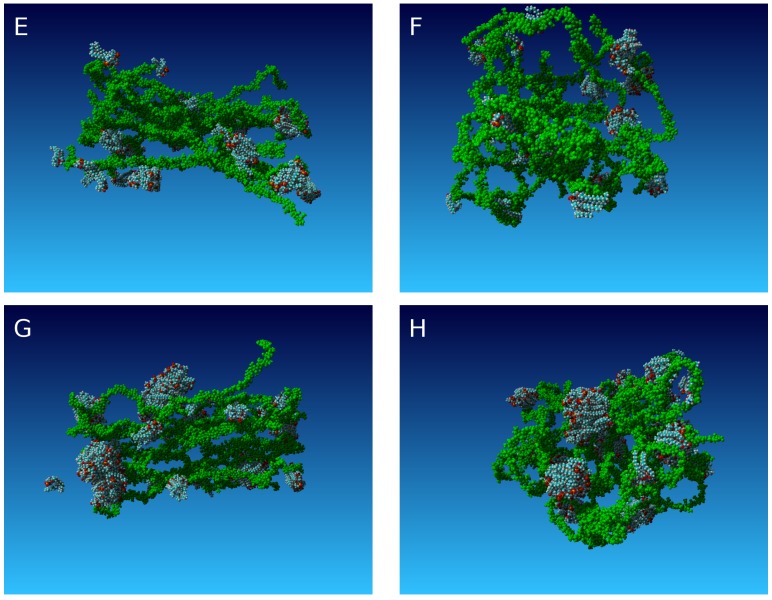
The structures of HA and PL at the end of the simulation: (**A**,**C**,**E**,**G**) results for a normal network; and (**B**,**D**,**F**,**H**) and for a pathological network. From (**A**,**B**) to (**G**,**H**), the number of phospholipids increases. Carbon atoms are depicted as blue; hydrogen as white; and nitrogen as yellow. Starting from the second row, HA is depicted as green for better visualization of PL orientation towards the HA network.

The final structures evaluated in this study are presented in Figure 2. From top to bottom, the concentration of lipids increased. Shown on the left hand side are highly organized inter-molecular HA networks typical of normal synovial fluid and, on the right side, unorganized, random coiled, intra-molecular HA networks typical of osteoarthritic synovial fluid.

**Figure 3:**
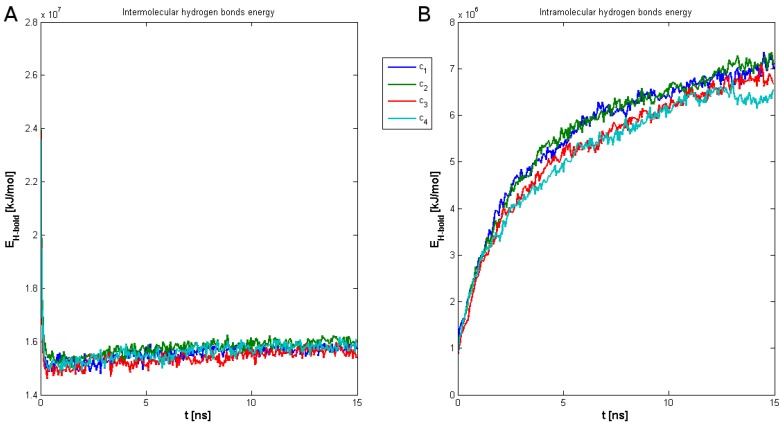
Total hydrogen bond energy in normal HA networks with increasing concentration of phospholipids: (**A**) intra-molecular H-bond energy is depicted; and (**B**) inter-molecular bond energy.

**Figure 4:**
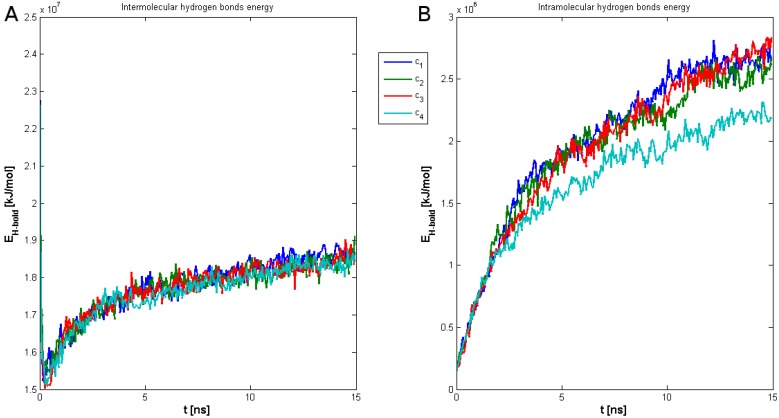
Total hydrogen bond energy in pathologic HA networks with increasing concentration of phospholipids: (**A**) intra-molecular H-bond energy is depicted; and (**B**) inter-molecular bond energy.

**Figure 5:**
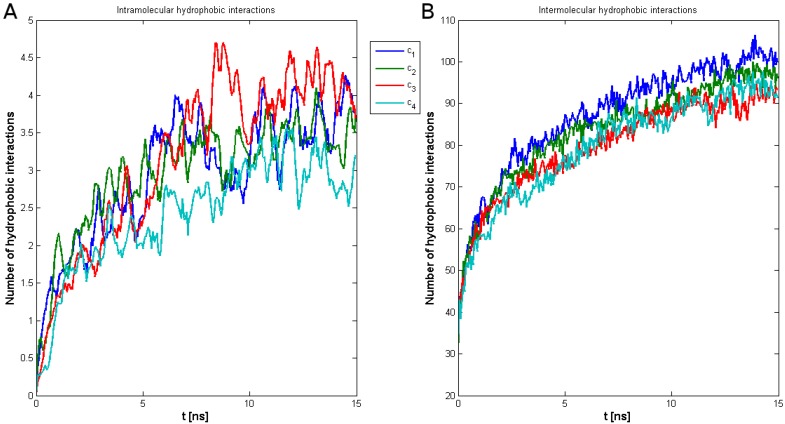
Total number of hydrophobic interactions for normal networks: (**A**) intra-molecular hydrophobic contacts are depicted; and (**B**) inter-molecular contacts.

**Figure 6:**
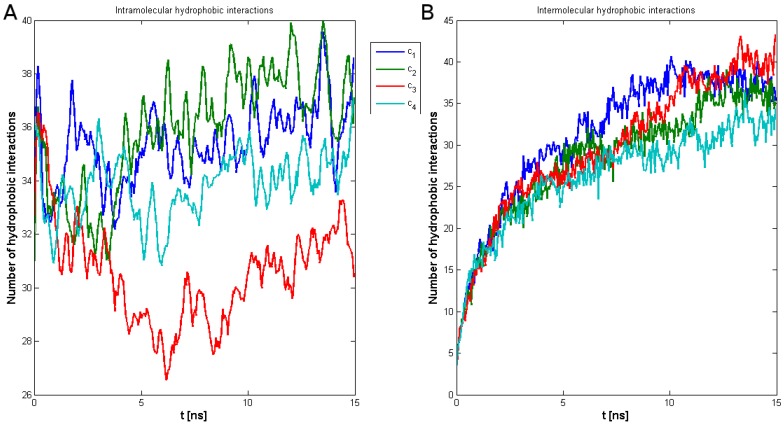
Total number of hydrophobic interactions for pathologic networks: (**A**) intra-molecular hydrophobic contacts are depicted; and (**B**) inter-molecular contacts.

One of the most important factors in both normal and abnormal molecular crosslinking of HA are hydrogen bond creation and hydrophobic contact sites between molecules. Figure 3 and Figure 4 show hydrogen bond energy inside HA networks. A significant difference in both structures is evident as more inter-molecular bonds exist in the case on the left hand side of Figure 2. The total number of H-bonds per unit time is the same for both cases. The number of hydrophobic interactions (Figure 5 and Figure 6) in both initial conditions behave in the same manner, namely without phospholipids, more hydrophobic interactions are evident. This finding is in agreement with experimental data showing that PL induces HA network hydrophobicity.

**Figure 7:**
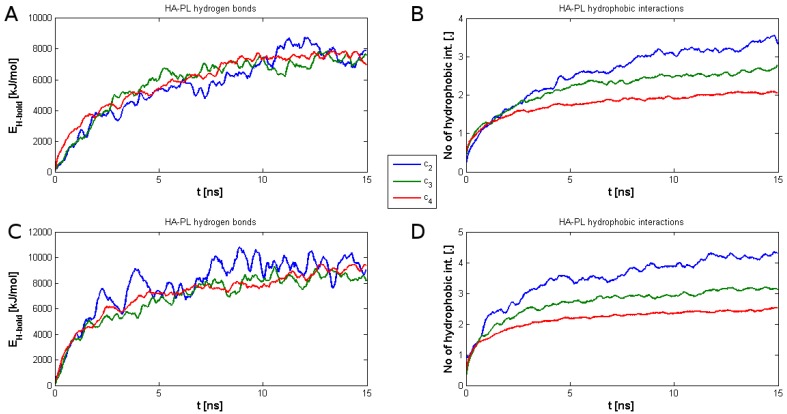
Normalized H-bond energy and hydrophobic interactions between HA and PL per one phospholipid: (**A**,**B**) inter-molecular networks are depicted; and (**C**,**D**) intra-molecular networks.

The hydrogen bond energy and hydrophobic interactions between HA and PL are presented in Figure 7 showing normalized values. PL tends to interact with HA more often in low PL concentrations as critical micelle concentration is not reached. Thus, it is energetically favorable to bind with HA.

It has been shown previously [12] that PL and HA interact in an energetically favorable manner, thus the higher penetration of PL into HA networks change its properties significantly; for example, the obtained structures have been found to be more flexible than pure HA solutions.

**Figure 8:**
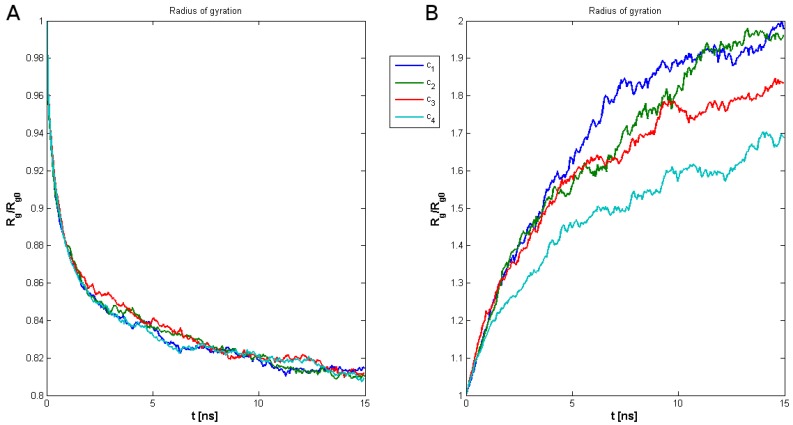
Radius of gyration of an average HA chain: (**A**) inter-molecular networks are depicted; and (**B**) intra-molecular networks.

Figure 8 presents the radius of gyration of average HA chains. The normal case is not dependent on PL concentration while the pathologic case is very sensitive to PL concentration. This is due to confinement caused by PL. The small impact of PL on the ’normal’ structure is a result of a worsening of phospholipid organization. The interplay between HA and PL is smaller and final chain length remains stable.

**Figure 9:**
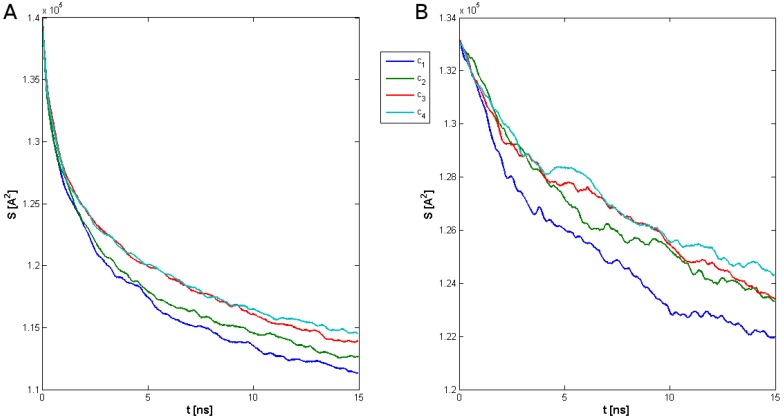
Solvent accessible surface of HA networks: (**A**) inter-molecular networks are depicted; and (**B**) intra-molecular networks.

The accessible molecular surface is presented in Figure 9, which shows how water molecules can penetrate HA networks. Normal HA networks repel water more rapidly as HA creates tighter networks whereas PL loosens HA interactions leaving space for water.

The mean square displacement of the system is time dependant as $MSD∼t^{\alpha }$. Values of α parameters have been shown in Table 1 and Table 2. HA in the normal case is more attached to the network, thus its diffusivity on average is smaller than in the pathological case. Lower concentrations PL tend to attach to HA and replace H-bonds resulting in more normal diffusion, however, at high PL concentrations, HA networks seem to be confined by PL as diffusion becomes abnormal.

## 3. Theoretical Considerations

The dynamics of large molecules have been formulated by many theories, but a full description requires quantum treatment that most often is not possible with traditional computing capacity. Therefore, approximations are often deployed, such as classical molecular dynamics, which does not obtain a level of quantum phenomena. Following this study, classical trajectories of large molecule segments in a landscape of potential energy is utilized. This approach provides approximate information about molecular dynamics and frequently explains many properties of large molecules. Sometimes, this approach yields information about the classical state dynamics of molecules that is too rich—such that the coarse graining method is better used in this scenario. In that method, a description is formulated in a language of probability, wherein the macromolecular system changes its state in a stochastic way. In this study, a classical mechanics approach is deployed as an in-silico experiment with quantities being resultant as experimental measurments. In this section, we establish the best statistical model that can describe the results is probability language. Statistical models that yield anomalous dynamics have a special place in the description of macromolecular systems which have a number of macromolecules co-existing. There are two popular stochastic models that introduce anomalous dynamics. The first is the fractional Brownian motion (FBM) and the second is continuous time random walk (CTRW). Mechanisms leading to anomalous dynamics for both processes are very different. In FBM, strong correlations between the increments of the process are responsible for ‘long memory’. In continuous time random walk, heavy tailed waiting times exist causing non-stationarity of process increments. The CTRW model has been used to describe protein dynamics [13,14]. An argument to use this model follows that the biological functional state of such macromolecules is represented by many substates that realize the same biological function [15]. Therefore, the following asymptotic form of waiting time distribution for this state can be obtained by

(1) $$\psi \left(t\right)∼C{\left(\frac{t}{\tau_{0}}\right)}^{-\beta },$$

where *C* and $\tau_{0}$ are a constant positive numbers.

The FBM was also used to decribe macromolecular systems. It is a special case of fractional Lévy α-stable motion (FLSM) with a self-similarity index *H* and a stability index α. For the fractional Brownian motion the stability index α=2 and the parameter d=H−1/2. This process may be described in terms of a generalized Langevin equation.

There exists a test to distinguish which model, CTWR, FBM or FLSM, is better in describing the evolution of macromolecules, and allow an indication of the most probable stochastic process responsible for observed non-Markovian evolution [16].

According to the work [16], we perform the sample mean square displacement test, which allows exclusion of either the FLSM or the CTRW model. Here, we use the following quantity

(2) $$M_{N}\left(\tau \right)=\frac{1}{N-\tau +1}\sum_{k=0}^{N-\tau }{\left(X_{k+\tau }-X_{k}\right)}^{2}.$$

Contrary to the classical mean square displacement, $M_{N}\left(\tau \right)$ is a random variable. If the stochastic process, underlying the recorded signal, is a fractional Lévy α-stable motion then

(3) $$M_{N}\left(\tau \right){∼}^{D}\tau^{2d+1},$$

where ${∼}^{D}$ denotes similarity in distributions. If the observed signal follows from the CTRW process then

(4) $$M_{N}\left(\tau \right){∼}^{D}\tau .$$

In our in-silico experiments, we obtained various geometrical and physical quantities that we treated as a stochastic variable. Frequently, we had only a few trajectories, therefore we decided to use the above mentioned test. 

**Figure 10:**
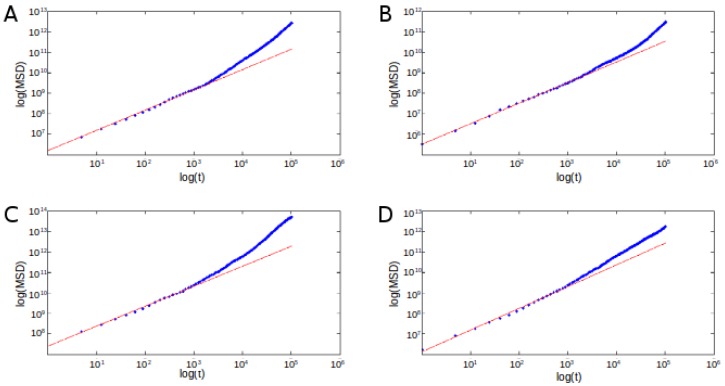
Linear fit to MSD, according to sample MSD method: $c_{1}$ (**A**,**C**); and $c_{4}$ (**B**,**D**); and (**A**,**B**) normal structure; and (**C**,**D**) abnormal structure.

The first quantity that we tested was motion of the macromolecules, calculated numerically according to formula

(5) $$MSD\left(\delta t\right)=\frac{1}{N}\sum_{i}^{N}|{\vec{r}}_{i}\left(\delta t\right)-\vec{r}\left(0\right){|}^{2},$$

where ${\vec{r}}_{i}$ is the position vector of an *i*-th atom. Results of this calculations are presented in the Figure 10, where we use two logarithmic scales.

According to the assumption of the sample MSD test τ<<N, we considered only several first values (20 points) of ln(τ). This regressions was done with coefficient of determination very close to one (see Table 3). We concluded that in the case of straight and parallel HA chains, the test does not reject the CTRW mechanism regardless of lipid presence. The value of the slope is close to one (0.988±0.032). Another situation is when HA chains are in a ball shape. For this case, we obtained the following values of the slopes: 0.975±0.023 for HA without phospholipids and 1.06±0.033 for HA with phospholipids. This result suggests that the shape of molecules play an important role in the selection the model underlying anomalous dynamics.

**Figure 11:**
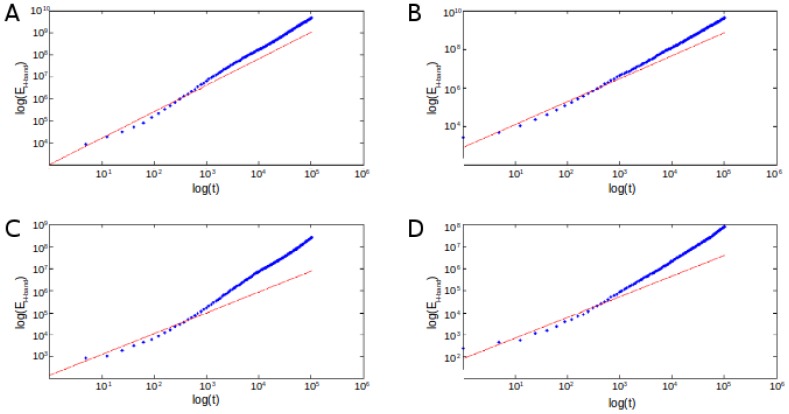
Linear fit to inter-molecular H-bond energies for HA according to sample MSD method. Two pictures in left column correspond to $c_{1}$ concentration and pictures in right column correspond to $c_{4}$ concentration. Two pictures in first row correspond to normal physiological structure. In the second row - pathological structure.

We obtained interesting results when hydrogen bond number is treated as a stochastic variable. The results are presented in Figure 11.

According to assumption τ<<N, we took only 10 points with ln(τ), where the coefficient of determination was close to one. These results suggest a disadvantage of continuous time random walk as an underlying microscopic mechanism observed results.

Parameter α from the table is directly connected with parameter 2d+1 in Equation (4) and there is the relation:

(6) α=2d+1.

The α number allows distinguishing between mentioned above processes. It is related to the fact that anomalous diffusion and mechanical matter relaxation of networks involving viscoelastic systems are conjugated phenomena [17], therefore there is no real suprise that the exponent of the form 2d+1 occurs as the characteristic value assigned to the fractional Brownian motion, Equation (4) and the α exponent included in it. Parameter β is a constant value from fitting and does not matter for this distinction.

We also performed another test that proved useful. The *p*-variation test is the statistical tool that is able to distinguish between stochastic mechanisms leading to anomalous diffusion. Similar to sample MSD, it is applied even to short one-trajectory series that follow from experiment. This test gives an explanation of origin of the anomalous diffusion. The basic concept of this test is the *p*-variation of a stochastic process X(t) performed in the time interval [0,T].

Discrete time-series following from this process was denoted by: $X_{0},X_{1},X_{2},\ldots ,X_{N}$. Its values appear in a discrete moments of time $t_{i}$, being binary division of [0,T] numbered by i=1,2,…,N. *p*-variation is defined by following formula:

(7) $$V_{p}\left(t_{j}\right)=\lim_{n\to \infty }V_{p}^{n}\left(t_{j}\right),$$

where $V_{p}^{n}\left(t_{i}\right)$ are given by:

(8) $$V_{p}^{n}\left(t_{j}\right)=\sum_{i}|X_{i2^{N-n}}-X_{\left(i-1\right)2^{N-n}}{|}^{p}$$

and index *i* fulfills following conditions: $0\leq \left(i-1\right)2^{M-n}$ and $i2^{M-n}\leq t_{j}.$ The number *n* can only have several values: 0,1,2,…,N. This equation was used to numerically obtain *p*-variation of analyzed processes.

We use this test on the quantity of molecules defined by Equation (5), in the case when CTRW seems not to be a proper model of phenomena. Quantity $V_{p}^{n}$, calculated according to Equation (8), as a function of *n* suggests that the proper model for time series is FLSM.

## 4. Discussion

In healthy synovial joint organ systems, the superficial active phospholipid layer remains intact and actively integrates tribological function toward efficient wear mitigation and tissue homeostasis [18,19]. This integration is cooperative and synergistic with the synovial fluid toward wound healing features [6] such that normal synovial fluid characteristics have been well tabulated [2]. Although the superficial active phospholipid layer is absent at sites of damaged articular cartilage, this layer can be reformed by surgical repair. This reformation now requires correlation to synovial fluid characteristics during and after surgical convalescence to determine which characteristics may persist due to various states of system pathogenesis. Therefore, this study was designed to evaluate HA in the presence of excess PL typically observed in osteoarthritic phenotypes [20,21], but modeled with an intact superficial active phospholipid layer characteristic of normal and/or surgically repaired tissue.

As depicted in Figure 2, two molecular constructs were studied, normal and osteoarthritic, which reflect different transition conditions between HA functionality at or around an intact superficial active phospholipid layer. While analyzing these spatial constructs, we observed an increase in PL concentration causes the HA network to be more hydrophobic and less crosslinked. The normal construct of HA networks was found to be characterized by higher stiffness, frequent chain-to-chain contact, less PL penetration, and small spatial variations in network structure. The osteoarthritic construct, however, was found to be a more elastic structure due to the modifications induced by excess PL and therefore less effective for lubrication.

**Figure 12:**
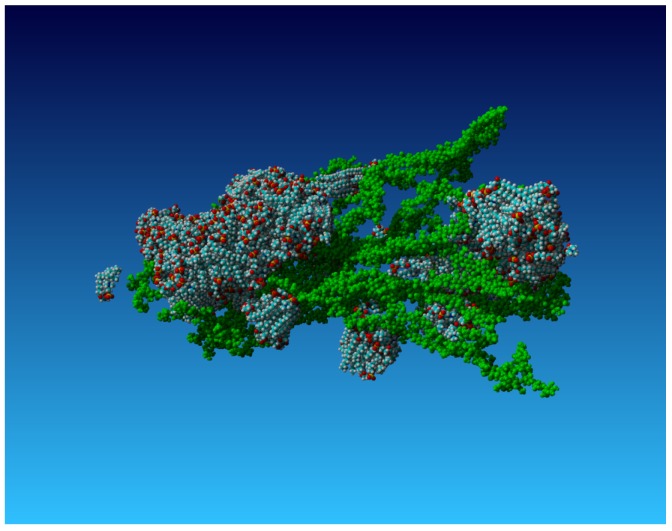
Final structure of 16 HA chains with PL in concentration $c_{5}=2\times c_{4}$. As one can see, PL can penetrate HA network and create micelle-like structures.

To explain these observations, we further analyzed the formed constructs and determined that hydrophobic reactions and hydrogen bonding in these structures may be substituted, or ‘penetrated’, by other reactions of the same kind, namely, between HA and PL. Penetration of normal constructs by PL was found to be possible, as presented in Figure 12, yet such penetration requires a very high PL concentration which occurs in the boundary lubrication zone, similar to the structures found in [22]. Because boundary lubrication regimes are typically when the superficial active phospholipid layer can act as a sacrificial layer for certain perturbation conditions, additional modeling is required to evaluate this unique situation and the various hysteresis effects of advancing and retreating lamellar and transitional bilayer structures, which is beyond the scope of this study.

Table 1 and Table 2 depict our findings of abnormal MSD value behaviors for the examined constructs. These anomalies are partly due to the PL penetration process into normal HA networks, which itself may result in HA chain disorder by releasing free HA chains into the joint cavity. For osteoarthritic constructs, on the other hand, substituting the connections within the HA network results in loosening the internal interactions within the network. These features illustrate the impact that the construct produce and the interaction of HA onto lubrication regimes, which, consequently, may provide some insight into pathogenesis evidenced by excess PL.

HA has versatile physiological properties which are dependent on its molecular length. Several mechanisms can provide various HA chain length species, both by biosynthesis and by differential degradation of larger HA polymers [12]. The influence that the superficial active phospholipid layer may have on this process should be further evaluated as well as their effect on overall lubrication mechanisms [23,24,25,26,27,28,29].

Our results show that this interaction is energetically preferable as it influences either HA constructs evaluated in this study and PL organization is mainly governed by HA length and PL concentration. Short HA chains are absorbed on PL vesicles, whereas long chains are surrounded with PL bilayers creating cylinder-like structures. Other results obtained by [30] showed that addition of DPPC to highly purified HA increased its flexibility, possibly due to competition between HA and PL for hydrophobic sites between chains.

The inter-molecular analysis presents a visualization of established network formation, whereas intra-molecular analysis involves HA network creation during pathological conditions. As presented in Figure 3 and Figure 4, the total inter-molecular H-bond energy is affected by number of phopholipids added. The introduction of hydrogen bonds in high-performance polymers has been deployed as a means to improve compressive strength [31]. Normal HA network display more inter-molecular H-bonds, thus behaving as a better perturbation dissipater throughout all functional forms. There is a smaller competition between HA and PL for H-bond creation, therefore the macromolecular forms can go through several cases. In least crowded PL case, there is a separation between both species or there are very little interactions between them reflected by $c_{1}$ and $c_{2}$ concentrations. HA creates dense networks which cannot be penetrated with PL. The penetration of PL into the networks is a factor responsible for proper functioning at other regimes. At higher concentrations, PL start, to create bilayer-like structures on networks (or in smaller scale connections between HA chains) which result in more flexible structures resulting in better response to the external load. Those structures can then better contribute to lowering friction force due to hydration lubrication mechanisms [32,33,34,35,36,37,38]. On the other hand, during osteoarthris pathogenesis, HA networks are much less viscous due to three-fold smaller intermolecular H-bond energy such that the addition of PL only generates more Newton-like fluid displaying pure lubricating properties. This finding can be also linked with the pH increases observed in osteoarthritic synovial fluid which resulting smaller $R_{g}$. As presented in Figure 8, there is tendency of HA chains to preserve their coiled structures when adding more phospholipids. Our results are in agreement with experimental data showing that PL increases hydrophobicity of HA (Figure 5 and Figure 6). In low concentrations of PL, a small effect on HA network is observed as PL binds to HA networks. However, even small concentrations of PL have an influence on water penetration of the network (Figure 9). There is also an effect on geometrical properties of HA chains. As presented in Figure 8, radius of gyration of single HA chain is affected as phospholipids are added due to crowding.

An additional simulation was performed to determine if the system displayed any significant changes under extremely high PL concentrations for the inter-molecular case. Figure 12 shows that, in high concentrations of PL, networks can be penetrated creating micelles inside HA networks. This would create space for another type of rolling friction inside HA networks. Roller HA:PL structures have been described before [12] in forms of HA:PL cylinders. This other type of formation would involve rolling friction of slightly different mechanisms [39,40]. Namely, HA networks could create the space for micelle involving interactions, as presented in [41,42,43,44]. Low friction could be then obtained due to hydration repulsion mechanisms between micelles or between HA and micelles [41,44]. Both forms could coexist simultaneously with similar mechanisms and effects. However to be useful for friction purposes, micelles require HA networks to normalize external load and prevent micelle collapse. Our MSD results (Figures Table 1 and Table 2) for all cases show that PL influence HA motion. The exponent a describes diffusivity of single HA chain show that on average the higher diffusivity is obtained for the intra-molecular case. In inter-molecular case, there is increase of MSD due to network penetration and lowering the number of H-bonds between chains allowing greater degrees of freedom. Above high PL concentrations, there is a confinement effect where HA has no space to expand. This is also the case for intra-molecular networks. The higher number of lipids decrease diffusivity of chains. There are still random process originating from the initial position of atoms which can cause freeing of networks. It has been shown that chondrocytes death is mediated by cartilage friction and wear [45]. Our results show that process of wear can be highly influenced by HA network condition. Well established HA network could possibly prevent system from degradation. However, once HA in degenerated SF, it acts more as a Newtonian fluid and cannot provide its viscoelastic properties.

Tests described above have shown that the geometrical form of HA has an important impact in choosing a proper statistical model to describe time evolution of of selected quantity of HA. We give argumentation that processes laying as a background of observed time series of molecular quantity have various origin.

## 5. Materials and Methods

**Figure 13:**
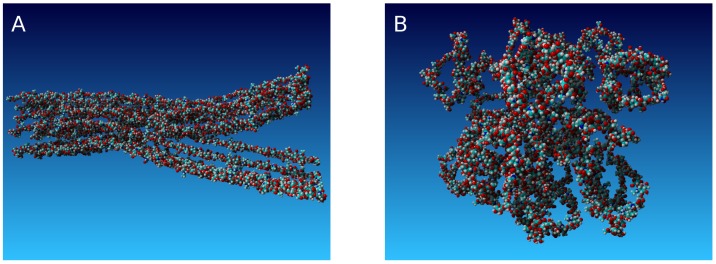
Initial structures of HA chains (water molecules and PL are not depicted): (**A**) a parallel (normal, inter-molecular) network is depicted; and (**B**) a coiled network (abnormal, intra-molecular) is depicted.

All-atom molecular dynamics simulations were performed using AMBER03 force fields to evaluate interactions between HA and PLs. The chosen force field has very general purposes and describes both molecules in a sufficient detail. HA and PL structures has been downloaded from PubChem (Open Chemistry DataBase). The lipid used for this study was dipalmityphosphatidocholine (DPPC). The HA molecule was modified to obtain longer chains by using YASARA structure software. The final length of HA was 25 nm and had a molecular mass of 10 MDa. All simulations were performed at the same conditions: temperature 310 K, pH = 7.0 and solvent 0.9% NaCl water solution. Sixteen HA chains were positioned in the water simulation container in two ways: parallel and coiled as presented in Figure 13.

Then, the different number of PLs were added to the solution in the following concentrations: $c_{1}=0$ (indicating pure HA solution), $c_{2}=5$, $c_{3}=10$ and $c_{4}=20\times 10^{-8}$ M, i.e., much higher than cmc of DPPC $c_{cmc}\approx 5\times 10^{-10}$ M. Final HA concentration was $c_{HA}=10^{-6}$ M—much higher than in syniovial fluid, however we focused on dense network of synovial fluid rather than overall concentration. The concentrations of PLs was adopted from others [20,21]. All systems were simulated for 15 ns ([3]), where a plateau was obtained. To describe a process of crosslinking, we used several variables described as follows. Radius of gyration $R_{g}$ is defined as the root mean square distance of the atoms from the center of mass as

(9) $$R_{g}=\sqrt{\frac{1}{N}\sum_{i=1}^{N}{\left(R_{i}-C\right)}^{2}}.$$

The hydrophobic interaction strength between hydrophobic atoms and hydrogen bond energy are calculated by the algorithm described previously [3].

Solvent accessible surface consisted of all the points that the center of the water probe (i.e., the nucleus of the oxygen atom in the water molecule) can reach while rolling over the solute. The procedure of calculating this variable has been presented previously in [46,47]. MSD (mean square displacement) is defined as $MSD\left(\delta t\right)=\frac{1}{N}\sum_{i}^{N}|{\vec{r}}_{i}\left(\delta t\right)-\vec{r}\left(0\right){|}^{2}$, where $\vec{r}$ is the position vector of an atom and δt is some time step.

## 6. Conclusions

The results presented in this study demonstrate that both initial conditions and PL concentration have a great impact on HA cross linking and the addition of excess PL typically observed during osteoarthritis pathogenesis increases hydrophobicity of HA networks. HA networks can behave as normal network, as they are more likely to interact with each other rather than with PL. For a more realistic study, we would have to turn toward coarse-grained models or hybrid models using both all-atom and coarse grained representations of HA and PL. Tests have shown that the anomalous dynamics of selected molecular quantities in a coarse–grained spatial scale can have different origins.

## Figures and Tables

**Table 1:** MSD of average HA chain for normal case fit parameters. Symbols: $c_{1}=0$ (indicating pure HA solution), $c_{2}=5$, $c_{3}=10$ and $c_{4}=20\times 10^{-8}$ M.

Case	$c_{1}$	$c_{2}$	$c_{3}$	$c_{4}$
$R^{2}$	0.9754	0.9634	0.9916	0.9783
α	0.826	0.8456	0.9073	0.7581

**Table 2:** MSD of average HA chain for abnormal case fit parameters. Symbols: $c_{1}=0$ (indicating pure HA solution), $c_{2}=5$, $c_{3}=10$ and $c_{4}=20\times 10^{-8}$ M.

Case	$c_{1}$	$c_{2}$	$c_{3}$	$c_{4}$
$R^{2}$	0.9792	0.9684	0.9626	0.9654
α	0.9247	0.9855	0.8715	0.8601

**Table 3:** Fitting parameters for data from Figure 10. Symbols: $c_{1}=0$ (indicating pure HA solution), $c_{2}=5$, $c_{3}=10$ and $c_{4}=20\times 10^{-8}$ M.

Case	$c_{1}$	$c_{2}$	$c_{3}$	$c_{4}$
$R^{2}$	0.9958	0.9987	0.9977	0.9962
α	0.9882	1.005	0.9748	1.06
β	6.201	5.506	7.400	6.121

## Abbreviations

The following abbreviations are used in this manuscript:

SF	Synovial Fluid
AC	Articular Cartilage
HA	Hyaluronic acid
PL	Phospholipids
OA	Ostheoarthritis
